# Influence of Obesity on Mid-term Revision Rates and Mortality After Elective Total Hip Arthroplasty—Analysis From the German Arthroplasty Registry (EPRD)

**DOI:** 10.1016/j.artd.2026.102029

**Published:** 2026-05-18

**Authors:** Jörg Lützner, Oliver Melsheimer, Alexander Grimberg, Klaus-Peter Günther, Cornelia Lützner, Arnd Steinbrück

**Affiliations:** a University Center for Orthopaedic, Trauma and Plastic Surgery, University Hospital Carl Gustav Carus, TU Dresden, Dresden, Germany; b German Arthroplasty Registry (EPRD), Berlin, Germany; c Orthopaedic Department, University Hospital Tübingen, Tübingen, Germany; d Center for Orthopaedic Surgery Augsburg (OCKA), Augsburg, Germany

**Keywords:** Obesity, BMI, Outcome, Hip, Arthroplasty, Replacement

## Abstract

**Background:**

Obesity has been associated with increased revision rates in total hip arthroplasty (THA). There is ongoing discussion as to whether this justifies postponing or denying THA to these patients. This study was initiated to determine the influence of obesity on revision rates and mortality after primary elective THA in Germany.

**Methods:**

The German Arthroplasty Registry (EPRD) provided data of 403,073 elective THAs with a valid body mass index (BMI) for analysis. Cumulative revision rates up to 7 years and 1-year mortality were calculated for BMI groups (nonobese, obesity grade 1, 2, and 3).

**Results:**

Increased revision rates with rising BMI were found. The main differences in revision rates appeared within the first postoperative months. This was most distinctive for periprosthetic joint infections during the first year after surgery (0.7% in nonobese, 1.3% in grade 1, 2.1% in grade 2, and 4.2% grade 3 obesity). Similar effects were observed in cementless and cemented stems. Revision rates in morbidly obese patients were not influenced by hospital caseload. Age- and sex-standardized mortality was lower than expected in most groups, but also increasing with rising BMI.

**Conclusions:**

Obesity increased the risk of revision following primary elective THA, particularly for periprosthetic joint infection in morbidly obese patients. However, the absolute risks of this potentially life-improving surgery may be acceptable for the patients affected. The potential risks and benefits should be weighed up individually for each patient, taking into account their BMI and any other risk factors they have.

**Level of Evidence:**

Level III, Therapeutic Study.

## Introduction

Obesity is increasing worldwide and will require relevant economic resources [1]. Already, 43% of adults in the United States and 30% in Australia are considered obese. Men are more frequently affected than women [2]. Obesity is a risk factor for hip osteoarthritis (OA). Consequently, there is a higher risk for the need of a hip arthroplasty at a younger age [3]. A cohort study of overweight and obese participants in Australia demonstrated that a weight reduction of at least 7.5% is required to reduce the need for a joint replacement [4].

Obese patients who undergo hip arthroplasty are at an increased risk of complications, especially morbidly obese patients with a body mass index (BMI) > 40 kg/m^2^. According to the German guideline, a BMI >40 kg/m^2^ is considered a relative contraindication for hip arthroplasty [5,6]. However, this does not mean an outright rejection of surgery, but rather that the associated risks and benefits must be carefully considered. In the United States, where the problem is even more prevalent than in Germany, a survey by the American Association of Hip and Knee Surgeons found that 73% of surgeons postpone arthroplasty surgery for patients with a BMI >40 kg/m^2^ until they have lost weight [7].

The currently available options for weight reduction (lifestyle and dietary adjustments, medication, and surgery) vary in effectiveness. Generally speaking, surgical measures are more effective. While a reduction in BMI of approximately 3 kg/m^2^ can be achieved through dietary counseling and exercise promotion, bariatric surgery achieves an improvement of approximately 14 kg/m^2^ [8]. Only around 20% of morbidly obese patients who were advised to lose weight before undergoing hip or knee arthroplasty achieved this goal [9,10]. The extent to which glucagon-like peptid-1 receptor analog outperform surgical therapy, particularly in terms of their short-term effectiveness, still remains to be investigated over the next years. Therefore, withholding a potentially life-improving arthroplasty due to a BMI >40 kg/m^2^ with otherwise fulfilled indication criteria is discussed critically [11].

This study was initiated to investigate the influence of obesity and its severity on mid-term revision rates and mortality in elective primary total hip arthroplasty (THA) in Germany. We hypothesized that higher BMI is associated with higher revision rates and mortality.

## Material and methods

The EPRD was founded by the German Society of Orthopaedics and Orthopaedic Surgery in cooperation with public health insurance companies (AOK, VdEK) and implant manufacturer (BVMed). The data collection at EPRD was approved by the institutional review board of the University of Kiel (ID 473/11, 22.11.2011). Written informed consent was obtained from all patients. More than 2 million arthroplasty procedures have been recorded in the EPRD since 2012 [12]. Both primary implantations and revision procedures are documented. Although participation in the EPRD is voluntary for hospitals and patients, around 70% of all hip and knee arthroplasties in Germany are recorded. If an arthroplasty is recorded in the registry, the follow-up with regard to revision and mortality is almost complete, as in addition to the entries made by the hospitals, information is also regularly transmitted by the participating health insurance companies [13,14]. Patients are followed up for revision, death, or amputation.

The EPRD currently tracks 874,648 primary hip and knee arthroplasties with valid BMI data, which have been recorded since 2017. All primary hip arthroplasties for which plausible information on height and weight was available were included in the analysis. As height and weight data entries cannot be verified in the EPRD, limit values were defined to avoid potential errors (height 1.20 m to 2.20 m, weight 30 kg to 180 kg, BMI 15 kg/m^2^ to 55 kg/m^2^). If values were outside these limits, the data were considered invalid and excluded from the analysis. Due to the higher risk of complications, nonelective THAs were excluded. Based on BMI, patients were categorized as nonobese (BMI <30 kg/m^2^), obese grade 1 (BMI 30 to <35 kg/m^2^), obese grade 2 (BMI 35 to <40 kg/m^2^), and obese grade 3 (BMI >40 kg/m^2^).

### Data analyses

Kaplan-Meier estimates were used to calculate the cumulative incidence of revisions (cumulative revision rate) for all causes. The cumulative revision rate was displayed separately for aseptic revisions and periprosthetic joint infection (PJI). Patients with incomplete follow-up and those who had no revision until the end of follow-up or death were “censored” at this point. Differences between cohorts were tested using the log-rank test stratified by age group and sex. If there were more than 2 cohorts, pairwise stratified log-rank tests were used with Bonferroni-Holm adjustment for multiple testing. The significance level was set at 0.05.

The standardized mortality ratio was calculated for the comparison of 1-year mortality. This was calculated as the quotient of the number of actual deaths within a year and the number of deaths expected in a year. To calculate the expected deaths, the 1-year mortality probability of patients of the same age and sex in Germany was determined for each patient using the mortality tables provided by the Federal Statistical Office and then totaled for all patients in the same group.

All analyses were carried out using the statistical software R (R Foundation for Statistical Computing, Vienna, Austria).

## Results

**Figure 1:**
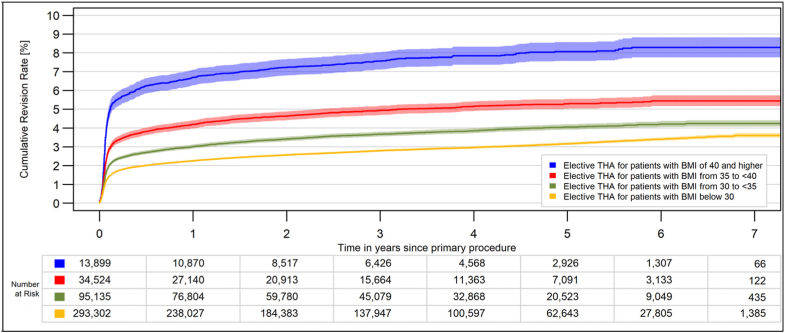
All-cause CRR for elective THA stratified for BMI groups (*P* < .0001). CRR, cumulative revision risk.

**Figure 2:**
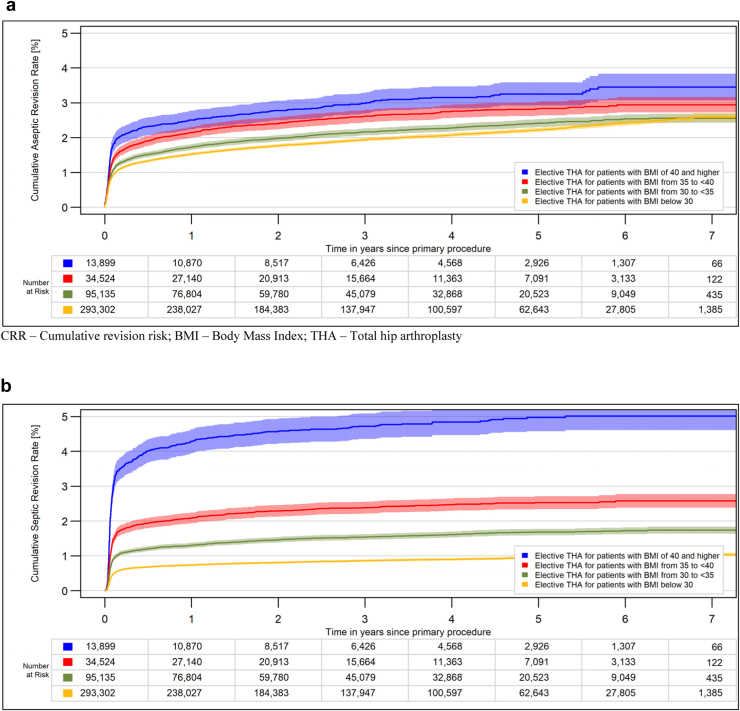
CRR for elective THA stratified for BMI groups. (a) Aseptic revisions (*P* < .0001). (b) Revisions for periprosthetic joint infection (*P* < .0001). CRR, cumulative revision risk.

A total of 403,073 elective THA were available for the analysis. The median age was 70 years (interquartile range: 61, 78), and 62.8% were women. The proportion of obese patients was 32.8% (grade 1, 2, and 3 obesity: 21.7, 7.8, and 3.2%). There was a clear correlation between BMI and revision rates (Fig. 1). The main differences in the revision rates appeared within the first postoperative months. While the differences between BMI groups for aseptic revisions were relatively small, the differences for PJI were substantial (Fig. 2). The 1-year PJI-related revision rates were as follows: 0.7% (95% confidence interval: 0.7, 0.8) for nonobese patients, 1.3% (1.3, 1.4) for grade 1 obesity, 2.1% (1.9, 2.2) for grade 2 obesity, and 4.2% (3.9, 4.6) for morbid obesity. The risk of a PJI within 1 year was 6.3 times higher for morbidly obese patients than for nonobese patients.

**Figure 3:**
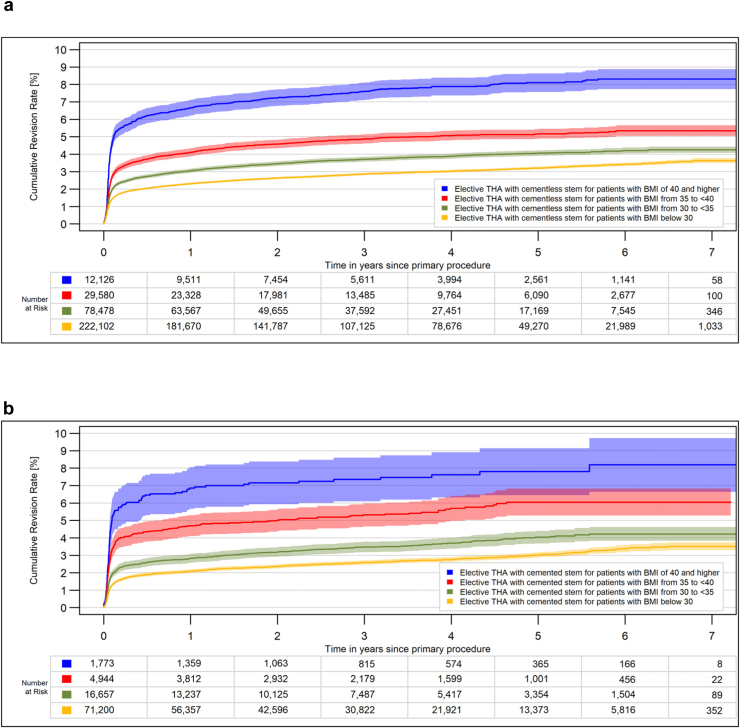
All-cause CRR for elective THA stratified for BMI groups. (a) Cementless stem (*P* < .0001). (b) Cemented stem (*P* < .0001). CRR, cumulative revision risk.

**Figure 4:**
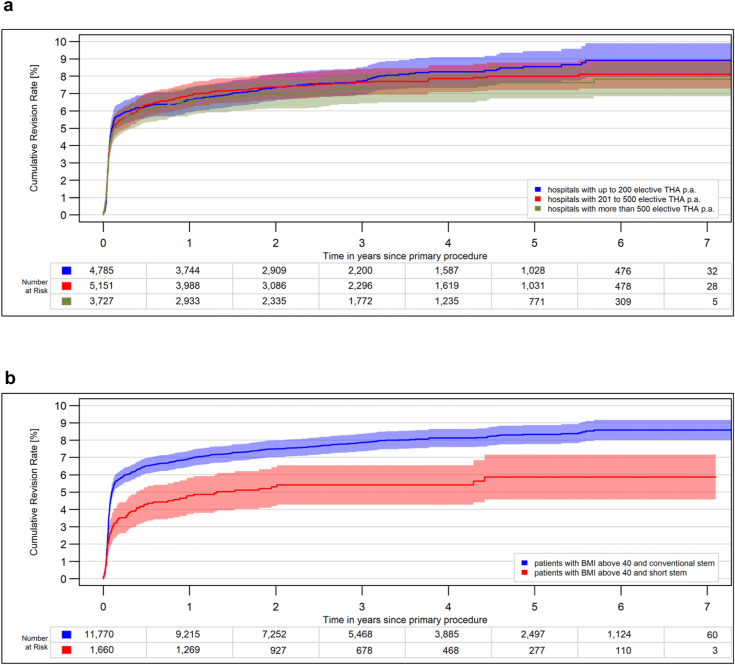
All-cause CRR for elective THA in morbidly obese patients (BMI >40). (a) Stratified for hospital caseload (*P* = .4). (b) Stratified for stem type (*P* = .01). CRR, cumulative revision risk; p.a., per annum.

Whether the stem fixation was cemented or cementless did not make any difference (Fig. 3). In morbidly obese patients, there was no difference in revision rates between hospitals with lower or higher caseloads (Fig. 4a). The use of a short stem was associated with lower revision rates in morbidly obese patients (Fig. 4b), probably because this type of stem is often used with a less invasive approach.

Mortality was lower in most BMI groups than would be expected after standardization by age and sex (standardized mortality ratio), although mortality increased with increasing BMI. However, mortality was slightly higher than expected in morbidly obese patients (Table 1).

**Table 1:** One-year mortality after elective total hip arthroplasty by BMI group unadjusted and as age- and sex-standardized mortality ratio and 95% confidence interval.

Patient group	Mortality unadjusted in %	SMR (95% CI)
BMI <30 kg/m^2^	1.4	0.51 (0.49-0.52)
BMI 30-35 kg/m^2^	1.1	0.53 (0.49-0.56)
BMI 35-40 kg/m^2^	1.1	0.68 (0.61-0.75)
BMI ≥ 40 kg/m^2^	1.3	1.08 (0.92-1.25)

CI, confidence interval; SMR, standardized mortality ratio.

## Discussion

Data from the EPRD demonstrated that the risk of revision surgery after elective primary hip arthroplasty increased with the degree of obesity. The risk was particularly high for PJI-related revisions. Although the 1-year mortality rate increased with rising BMI, it was generally lower than expected except for morbidly obese patients.

Systematic reviews and meta-analyses have clearly shown an association between obesity and higher rates of complications in THA. In a study of 161,785 patients, DeMik et al. [15] found an increased risk of complications, particularly periprosthetic infections and reoperations, with increasing BMI. The risk was significantly higher with THA than with total knee arthroplasty. Onggo et al. [16] analyzed 67 studies with 2.2 million THA. Compared to patients with a BMI of less than 30 kg/m^2^, obese patients had a significantly higher risk of complications, particularly PJI. These risks were even higher in morbidly obese patients, with a BMI of 40 kg/m^2^ or more. Another systematic review analyzed the influence of BMI on periprosthetic infections [17]. It revealed that the risk of PJI increased exponentially from a BMI of >24 kg/m^2^. This increase was approximately 1.5 times more pronounced for THA than total knee arthroplasty.

Unlike the EPRD, most international arthroplasty registries do not explicitly describe the influence of BMI on revision rates in their annual reports. The Australian registry (Australian Orthopaedic Association National Joint Registry) showed a similar increase in revision rates for conventional THA as the EPRD, particularly within the first 3 months (3.15-fold increase in grade 3 obesity compared to normal weight).

The proportion of morbidly obese patients (BMI >40 kg/m^2^) undergoing hip arthroplasty varies internationally. While it is only 0.6% for THA in Sweden [18], it is higher in Australia (6.0%) [19] and the United States (6.8%) [20] than in Germany (3.2%). This is noteworthy considering that, according to the Organisation for Economic Co-operation and Development, the Swedish population has a similar obesity rate (15%) to the German population (17%) [2]. The higher proportion of arthroplasty procedures performed on morbidly obese patients in Australia and the United States is easily explained by the higher prevalence of obesity in these populations. Overall, a lower proportion of morbidly obese patients undergo hip arthroplasty than knee arthroplasty. This may be because obesity has a greater influence on the development of knee OA than hip OA. However, the higher risk of complications associated with hip arthroplasty in morbidly obese patients may result in more restrictive indications.

This raises the question of whether arthroplasty surgery should be refused based on BMI. Apart from an increased risk of complications, obese patients appear to experience the same benefits from hip arthroplasty as nonobese patients in terms of improved function and quality of life. Although the Australian Orthopaedic Association National Joint Registry showed poorer overall preoperative and postoperative scores for obese patients, the gains from hip arthroplasty were comparable [21]. The Swedish Hip Arthroplasty Registry revealed that primary THA patients who were underweight, overweight, or obese had worse preoperative and postoperative scores for hip pain and health-related quality of life (Euroquol questionnaire, version 5D), but experienced a comparable improvement after surgery [22]. There is clear evidence that a high BMI increases the risk of complications after THA, but there is currently no evidence that weight loss can reduce this risk [23]. In contrast, effective weight loss through bariatric surgery appears to increase the risk of complications, at least in the short term [8,24]. A recent review based on the current literature [8] concluded that, although obese patients are at an increased risk of complications, they can still benefit greatly from “life-changing” arthroplasty surgery and should not be denied this option. This corresponds with the recommendation in the current joint guideline from the American College of Rheumatology and the American Association of Hip and Knee Surgeons that a THA should not be postponed due to certain BMI values [25]. However, the accompanying text emphasizes that this recommendation is case-specific and that the evidence base is considered to be limited. According to current data, postponing surgery in order to lose weight does not reduce the risk. Furthermore, weight reduction is not successful in the majority of patients [9,10].

A case-control study of 13,223 THA in the United States showed that clinical outcomes and complications following THA performed by high-volume surgeons (among the top 25% of annual case numbers) were independent of BMI [26]. The authors concluded that morbidly obese patients would benefit from being referred to a high-volume surgeon. This effect was not evident in the EPRD data. Here, the risk of revision surgery increased with rising BMI, regardless of the hospital caseload. However, the number of cases performed by individual surgeons is not yet recorded in the EPRD.

We acknowledge some limitations. In addition to BMI, other diseases commonly associated with obesity, such as diabetes mellitus and nicotine abuse, are also known risk factors for complications. Information on these diseases was not available for this study and may have influenced the results. As the EPRD is voluntary, not all procedures in Germany are recorded. However, since around 70% of all arthroplasty implantations in Germany are recorded in the EPRD, the data are still considered representative. The near-complete follow-up of patients registered in the EPRD yields highly reliable data on the German healthcare landscape. However, the registry only contains data on revisions and mortality. As some revisions are only recorded in the International Classification of Diseases/International Classification of Procedures in Medicine reports from health insurance companies, no further details are available for these cases, and it is only possible to distinguish between aseptic and septic revisions. Nonvalid data were excluded from the analysis. The EPRD is a relatively new registry with data collection running for 12 years. Therefore, only medium-term follow-up data are currently available. It is uncertain whether the higher weight load of obese patients will eventually lead to an increase in aseptic loosening in the long term. It has not been conclusively clarified if the BMI is the appropriate criterion for assessing obesity. Nevertheless, it is currently the most suitable and internationally comparable parameter available.

## Conclusions

Obesity increased the risk of revisions following primary elective THA, particularly for PJI in morbidly obese patients. However, the absolute risks of this potentially life-changing surgery may be acceptable to affected patients. The potential risks and benefits should be weighed individually for each patient, taking into account their BMI and any other risk factors they have.

## CRediT authorship contribution statement

**Jörg Lützner:** Writing – review & editing, Writing – original draft, Project administration, Conceptualization. **Oliver Melsheimer:** Writing – review & editing, Visualization, Methodology, Formal analysis, Data curation, Conceptualization. **Alexander Grimberg:** Writing – review & editing, Project administration, Methodology, Conceptualization. **Klaus-Peter Günther:** Writing – review & editing, Supervision, Conceptualization. **Cornelia Lützner:** Writing – review & editing, Visualization, Validation. **Arnd Steinbrück:** Writing – review & editing, Supervision, Project administration, Conceptualization.

## Conflicts of interest

A. Steinbrück is on the speakers bureau/paid presentations for Johnson & Johnson and is a scientific officer for German Arthroplasty Registry (EPRD). J. Lützner is on the speakers bureau/paid presentations for Aesculap and Enovis; is a paid consultant for Aesculap and Enovis; received research support from Aesculap, Enovis, Link, Smith & Nephew, and Zimmer Biomet as a Principal Investigator; and is a board member/committee appointments for ISAR. K.-P. Günther is on the speakers bureau/paid presentations for MedUpdate and Zimmer Biomet; received research support from Zimmer Biomet as a Principal Investigator; is a member of the medical/orthopaedic publications editorial/governing board at OU Up2Date and OrthoTrauma Update; and is a board member/committee appointments for German Society of Orthpaedics and Orthopaedic Surgery (DGOOC), German Arthroplasty Registry (EPRD), German Society for Orthopaedics and Traumatology (DGOU), European Hip Society (EHS), and European Federation of Orthopaedics and Traumatology (EFORT); all other authors declare no potential conflicts of interest.

For full disclosure statements refer to https://doi.org/10.1016/j.artd.2026.102029.

## Appendix A Supplementary data

Conflict of Interest Statement for Grimberg

Conflict of Interest Statement for Steinbrück

Conflict of Interest Statement for Jörg Lützner

Conflict of Interest Statement for Günther

Conflict of Interest Statement for Melsheimer

Conflict of Interest Statement for Cornelia Lützner

## References

[1] Carender C.N., Glass N.A., DeMik D.E., Elkins J.M., Brown T.S., Bedard N.A. (2022). Projected prevalence of obesity in primary total hip arthroplasty: how big will the problem get?. J Arthroplasty.

[2] Organisation for Economic Co-operation and Development (OECD) (2023). Health at a Glance 2023. http://10.1787/7a7afb35-en.

[3] Gandhi R., Wasserstein D., Razak F., Davey J.R., Mahomed N.N. (2010). BMI independently predicts younger age at hip and knee replacement. Obesity (Silver Spring).

[4] Jin X., Gibson A.A., Gale J., Schneuer F., Ding D., March L. (2021). Does weight loss reduce the incidence of total knee and hip replacement for osteoarthritis?—A prospective cohort study among middle-aged and older adults with overweight or obesity. Int J Obes.

[5] (2019). Deutsche Gesellschaft für Orthopädie und Unfallchirurgie e.V. (DGOU). S3-Leitlinie Evidenz- und konsensbasierte Indikationskriterien zur Hüfttotalendoprothese bei Coxarthrose (EKIT-Hüfte) (AWMF-Registernummer 187-001). https://www.awmf.org/leitlinien/detail/ll/187-001.html.

[6] Lützner C., Deckert S., Günther K.P., Postler A.E., Lützner J., Schmitt J. (2022). Indication criteria for total hip arthroplasty in patients with hip osteoarthritis-recommendations from a German consensus initiative. Medicina (Kaunas).

[7] Abdel M.P., Carender C.N., Berry D.J. (2023). Current practice trends in primary hip and knee arthroplasties among members of the American Association of hip and knee surgeons. J Arthroplasty.

[8] Blankstein M., Browne J.A., Sonn K.A., Ashkenazi I., Schwarzkopf R. (2023). Go big or Go home: obesity and total joint arthroplasty. J Arthroplasty.

[9] Reeves R.A., Hefter G.D., Pellegrini V.D., Drew J.M., Barfield W.R., Demos H.A. (2021). The fate of morbidly Obese patients with joint pain: a retrospective study of patient outcomes. J Arthroplasty.

[10] Shapiro J.A., Narayanan A.S., Taylor P.R., Olcott C.W., Del Gaizo D.J. (2020). Fate of the morbidly Obese patient who is denied total joint arthroplasty. J Arthroplasty.

[11] Carender C.N., Fruth K.M., Lewallen D.G., Berry D.J., Abdel M.P., Bedard N.A. (2025). Obesity and primary total knee arthroplasty: the absolute versus relative risk of periprosthetic joint infection at 15 years. J Arthroplasty.

[12] The German Arthroplasty Registry (EPRD) (2025). Annual Report 2024. https://www.eprd.de/fileadmin/user_upload/Dateien/Publikationen/Berichte/AnnualReport2024-Web_2025-03-27_F.pdf.

[13] Grimberg A.W., Steinbrück A. (2023). 10 years of the German Arthroplasty Registry-EPRD: what has been achieved?. Orthopadie.

[14] Jansson V., Grimberg A., Melsheimer O., Perka C., Steinbruck A. (2019). Orthopaedic registries: the German experience. EFORT Open Rev.

[15] DeMik D.E., Bedard N.A., Dowdle S.B., Elkins J.M., Brown T.S., Gao Y. (2018). Complications and obesity in arthroplasty—a hip is not a knee. J Arthroplasty.

[16] Onggo J.R., Onggo J.D., de Steiger R., Hau R. (2020). Greater risks of complications, infections, and revisions in the obese versus non-obese total hip arthroplasty population of 2,190,824 patients: a meta-analysis and systematic review. Osteoarthritis Cartilage.

[17] Zhong J., Wang B., Chen Y., Li H., Lin N., Xu X. (2020). Relationship between body mass index and the risk of periprosthetic joint infection after primary total hip arthroplasty and total knee arthroplasty. Ann Transl Med.

[18] The Swedish Arthroplasty Register (SAR) (2025). Annual report 2024. https://registercentrum.blob.core.windows.net/sar/r/Swedish-Arthroplasty-Register-Annual-report-2024-ENG-CHsgLK06p.pdf.

[19] Australian Orthopaedic Association National Joint Replacement Registry (AOANJRR) (2024). Annual report. Hip, Knee and Shoulder Arthroplasty. https://aoanjrr.sahmri.com/documents/10180/1798900/AOANJRR+2024+Annual+Report.pdf/9d0bfe03-2282-8fc8-a424-b8d9abb82b1f?t=1727666185313.

[20] American Joint Replacement Registry (AJRR) (2022). Annual report 2022. The Ninth annual Report of the AJRR on Hip and Knee Arthroplasty. https://connect.registryapps.net/hubfs/PDFs%20and%20PPTs/2022%20AJRR%20Annual%20Report.pdf.

[21] Mulford J.S., Ackerman I., Holder C., Cashman K.S., Graves S.E., Harris I.A. (2023). The association between body mass index and patient-reported outcome measures before and after primary total hip or knee arthroplasty: a registry. ANZ J Surg.

[22] Mukka S., Rolfson O., Mohaddes M., Sayed-Noor A. (2020). The effect of body mass index class on patient-reported health-related quality of life before and after total hip arthroplasty for osteoarthritis: registry-based cohort study of 64,055 patients. JBJS Open Access.

[23] LaValva S.M., Grubel J., Ong J., Chiu Y.-F., Lyman S., Mandl L.A. (2024). Is preoperative weight reduction in patients who have body mass Index≥ 40 associated with lower complication rates after primary total hip arthroplasty?. J Arthroplasty.

[24] Oettl F.C., Weinblatt A.I., Lan R., Gutierrez Naranjo J.M., Gonzalez A.G., Ichinose R. (2025). A history of bariatric surgery negatively affects the rate of 90-Day complications of elective primary total joint arthroplasty: a comparative analyses of 2,979 patients. J Arthroplasty.

[25] Hannon C.P., Goodman S.M., Austin M.S., Yates A., Guyatt G., Aggarwal V.K. (2023). 2023 American College of Rheumatology and American Association of hip and knee surgeons clinical practice guideline for the optimal timing of elective hip or knee arthroplasty for patients with symptomatic moderate-to-severe osteoarthritis or advanced symptomatic osteonecrosis with secondary arthritis for whom nonoperative therapy is ineffective. J Arthroplasty.

[26] Ashkenazi I., Thomas J., Lawrence K.W., Meftah M., Rozell J.C., Schwarzkopf R. (2024). The impact of obesity on total hip arthroplasty outcomes when performed by high-volume Surgeons-A propensity matched analysis from a high-volume urban center. J Arthroplasty.

