# Supplementary material for: Influence of Obesity on Mid-term Revision Rates and Mortality After Elective Total Hip Arthroplasty—Analysis From the German Arthroplasty Registry (EPRD)
Source: Arthroplast Today. 2026 May 18;39:102029. doi: 10.1016/j.artd.2026.102029 (PMC13202534; doi:10.1016/j.artd.2026.102029)
Supplement: Conflict of Interest Statement for Steinbrück [file mmc2.pdf]

INDIVIDUAL CONFLICT OF INTEREST STATEMENT

American Association of Hip and Knee Surgeons

(Adopted from the American Academy of Orthopaedic Surgeons disclosure statement)

The following form **must be filled out completely and submitted by each author (example, 6 authors, 6 forms). All items require a response. If there is no relevant disclosure for a given item, enter "*None.*"**

Manuscript Title: Influence of obesity on revision rates and mortality after elective Total Hip Arthroplasty - Analysis from the German Arthroplasty Registry (EPRD)

1. Royalties from a company or supplier (The following conflicts were disclosed)

None

2. Speakers bureau/paid presentations for a company or supplier (The following conflicts were disclosed)

JohnsonJohnson

3A. Paid employee for a company or supplier (The following conflicts were disclosed)

None

3B. Paid consultant for a company or supplier (The following conflicts were disclosed)

None

3C. Unpaid consultants for a company or supplier (The following conflicts were disclosed)

None

4. Stock or stock options in a company or supplier (The following conflicts were disclosed)

None

5. Research support from a company or supplier as a Principal Investigator (The following conflicts were disclosed)

None

6. Other financial or material support from a company or supplier (The following conflicts were disclosed)

None

7. Royalties, financial or material support from publishers (The following conflicts were disclosed)

None

8. Medical/Orthopaedic publications editorial/governing board (The following conflicts were disclosed)

None

9. Board member/committee appointments for a society (The following conflicts were disclosed)

Scietific officer German Arthroplasty Registry (EPRD)

Each author must sign AND print or type his/her name, date and submit a separate form

In addition, one BLINDED Conflict of Interest form (no author names used) should be submitted per manuscript with all author disclosures.

Arnd Steinbrück	AS	07/17/2025
Author Name (Print or Type)	Author Signature	Date
