# Supplementary material for: Influence of Obesity on Mid-term Revision Rates and Mortality After Elective Total Hip Arthroplasty—Analysis From the German Arthroplasty Registry (EPRD)
Source: Arthroplast Today. 2026 May 18;39:102029. doi: 10.1016/j.artd.2026.102029 (PMC13202534; doi:10.1016/j.artd.2026.102029)
Supplement: Conflict of Interest Statement for Günther [file mmc4.pdf]

INDIVIDUAL CONFLICT OF INTEREST STATEMENT

American Association of Hip and Knee Surgeons

(Adopted from the American Academy of Orthopaedic Surgeons disclosure statement)

Manuscript Title: Influence of obesity on revision rates and mortality after elective Total Hip Arthroplasty - Analysis from the German Arthroplasty Registry (EPRD)

1. Royalties from a company or supplier (The following conflicts were disclosed)

None

2. Speakers bureau/paid presentations for a company or supplier (The following conflicts were disclosed)

MedUpdate, ZimmerBiomet

3A. Paid employee for a company or supplier (The following conflicts were disclosed)

None

3B. Paid consultant for a company or supplier (The following conflicts were disclosed)

None

3C. Unpaid consultants for a company or supplier (The following conflicts were disclosed)

None

4. Stock or stock options in a company or supplier (The following conflicts were disclosed)

None

5. Research support from a company or supplier as a Principal Investigator (The following conflicts were disclosed)

Zimmer Biomet

6. Other financial or material support from a company or supplier (The following conflicts were disclosed)

None

7. Royalties, financial or material support from publishers (The following conflicts were disclosed)

None

8. Medical/Orthopaedic publications editorial/governing board (The following conflicts were disclosed)

OU Up2Date, OrthoTrauma Update

9. Board member/committee appointments for a society (The following conflicts were disclosed)

German Society of Orthpaedics and Orthopaedic Surgery (DGOOC), German Arthroplasty Registry (EPRD), German Society for Orthopaedics and Traumatology (DGOU), European Hip Society (EHS), European Federation of Orthopaedics and Traumatology (EFORT)

Klaus-Peter Günther K. Günther Date: 12.08.2025
